# Antioxidant and Neuroprotective Potential of the Brown Seaweed *Bifurcaria bifurcata* in an in vitro Parkinson’s Disease Model

**DOI:** 10.3390/md17020085

**Published:** 2019-02-01

**Authors:** Joana Silva, Celso Alves, Rafaela Freitas, Alice Martins, Susete Pinteus, Joana Ribeiro, Helena Gaspar, Amparo Alfonso, Rui Pedrosa

**Affiliations:** 1 MARE—Marine and Environmental Sciences Centre, ESTM, Instituto Politécnico de Leiria, 2520-641 Peniche, Portugal; celso.alves@ipleiria.pt (C.A.); rafaela.freitas@ipleiria.pt (R.F.); alice.martins@ipleiria.pt (A.M.); susete.pinteus@ipleiria.pt (S.P.); joana.c.ribeiro@ipleiria.pt (J.R.); hmgaspar@fc.ul.pt (H.G.); 2 Department of Pharmacology, Faculty of Veterinary, University of Santiago de Compostela, 27002 Lugo, Spain; amparo.alfonso@usc.es; 3 BioISI-Biosystems & Integrative Sciences Institute, Faculty of Sciences, University of Lisboa, Campo Grande, C8, 1749-016 Lisboa, Portugal

**Keywords:** 6-hydroxydopamine, seaweeds, SH-SY5Y cells, neuroprotection, mitochondrial membrane potential, Caspase-3 activity, eleganolone, eleganonal, neurodegenerative diseases

## Abstract

*Bifurcaria bifurcata* is a marine brown seaweed mainly found on the Atlantic coast. Herein, we report the antioxidant and neuroprotective activities of seven fractions (F1–F7) obtained by normal phase chromatography from the *B. bifurcata* dichloromethane extract, as well as of its two major isolated diterpenes. Total phenolic content of fractions was determined by the Folin–Ciocalteu method, while antioxidant activity was evaluated by the DPPH, ORAC, and FRAP assays. Neuroprotective effects were evaluated in a neurotoxic model induced by 6-hydroxydopamine (6-OHDA) in a human neuroblastoma cell line (SH-SY5Y), while the mechanisms associated to neuroprotection were investigated by the determination of mitochondrial membrane potential, H_2_O_2_ production, Caspase-3 activity, and by observation of DNA fragmentation. Fractions F4 and F5 exhibited the best neuroprotective and antioxidant activities, respectively. F4 fraction prevented changes in mitochondrial potential, and induced a reduction of H_2_O_2_ levels production and an increase in cell viability, suggesting that it may contain multi-target compounds acting on different pathways. Hence, this fraction was subjected to purification steps, affording the known diterpenes eleganolone and eleganonal. Both compounds exhibited antioxidant potential, being interesting candidates for further neuroprotective studies.

## 1. Introduction

Parkinson’s disease (PD) is a common neurodegenerative disease which affects the movement of aged population, and is characterized by the accumulation of Lewy bodies, loss of dopaminergic neurons in the *substantia nigra* of *pars compacta* (SNpc), and depletion of the neurotransmitter dopamine (DA) [1,2]. In recent years, many researchers have been focused on the prospection of new compounds from natural origin, since nature has proven to be a unique source of compounds with biomedical applications [3,4,5]. These metabolites may be intermediates or end-products of metabolism and are classified as primary metabolites which are essential for life, while the secondary ones participate in the natural defense of organisms [6]. The marine ecosystem can be regarded as an unlimited source of new secondary metabolites with unmatched chemical structures that can inspire the discovery of new drugs for the treatment of several pathologies, including PD [7,8]. Amongst the various marine organisms, seaweeds are a rich source of bioactive compounds that are not found in other organisms, exhibiting diverse biological activities, such as antioxidant, antitumor, antimicrobial, neuroprotector, antiviral, and anti-inflammatory, among others [9,10,11]. Seaweeds are one of the most accessible marine resources in the coastal zone, being classified in green, red, and brown algae [12]. Among the various species of macroalgae, only a limited number has been the subject of several studies because of their rich chemical composition and diversity of biological activity or health benefits, namely brown algae [13]. These algae have shown the ability to produce a wide variety of secondary metabolites with unique structures exhibiting various functionalities [14], particularly the algae *Bifurcaria bifurcata*. It can be found on the Atlantic coast (France, Spain, and Portugal) or on the west coast of Ireland [15], and has in their organic extracts several linear diterpenes rarely found in other brown species, [16]. Effectively, various in vitro biological effects of *B. bifurcata* extracts have been described, e.g., antioxidant, antimicrobial, and antitumor activities, which were attributed to the presence of these diterpenes [17,18,19,20]. Recently, a study reported by Alves et al. [19] demonstrated the bioactive potential of the methanolic and dichloromethane extracts of *B. bifurcata* regarding their antioxidant and antitumor properties, highlighting the importance of the isolation and identification of compounds that could be responsible for the bioactivities.

Pursuing our work on the study of the therapeutic potential of seaweeds collected in the Peniche coast, Portugal, we report now the antioxidant and neuroprotective activities of fractions obtained from *B. bifurcata* dichloromethane extract. In addition, the isolation and characterization of compounds isolated from the most bioactive fraction is presented, aiming to support and to rationalize the mechanisms of neuroprotection herein reported.

## 2. Results and Discussion

### 2.1. Antioxidant Activity of B. bifurcata

In the evaluation of the antioxidant activity of dichloromethane extract from *B. bifurcata* and derived fractions (F1–F7), different approaches have been outlined, namely, total phenolic content (TPC) determination by the Folin–Ciocalteu method, 2,2-diphenyl-1-picrylhydrazyl radical scavenging (DPPH) assay, oxygen radical absorbance capacity (ORAC) method, and ferric reducing antioxidant power (FRAP) assay. The results obtained are shown in Table 1.

F5 fraction presented the highest phenolic content (44.14 ± 1.30 mg gallic acid equivalents (GAE)/g extract), when compared to other fractions. Regarding the DPPH radical scavenging ability, F5 and F7 fractions presented the highest potential, being F5 the most potent one with an IC_50_ of 49.73 µg/mL (31.56–78.56) for a 95% confidence interval. In the ORAC method, F5 and F7 fractions showed the highest antioxidant activity, 4469.14 ± 147.07 µmol of Trolox/g extract, and 2861.25 ± 38.92 µmol of Trolox/g extract, respectively. F5 fraction also revealed to be effective in reducing ferric ions (1128.20 ± 20.25 µM FeSO_4_/g extract) when compared with other fractions (Table 1). 

**Figure 1:**
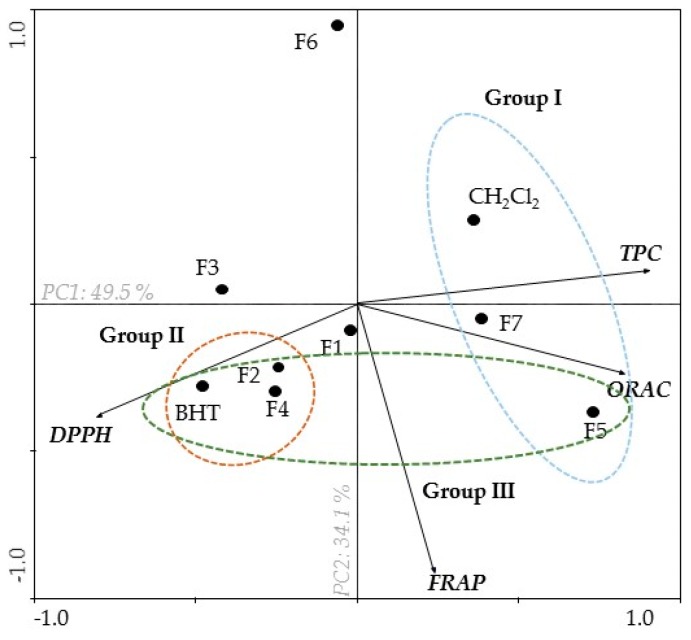
Principal component analysis (PCA) of total phenolic content (TPC) and antioxidant activities (DPPH, ORAC, FRAP) of *B. bifurcata* dichloromethane extract (CH_2_Cl_2_), fractions (F1–F7), and standard (BHT).

The main components analysis (PCA) carried out in this study allowed us to relate all the methods used to evaluate the antioxidant capacity (DPPH, ORAC, and FRAP) and total phenolic content (TPC). It is possible to observe the clear arrangement of three groups that differ according to their antioxidant activity (Figure 1). 

As referred to above, this PCA was carried out to obtain an overview of the similarities and differences among the seven fractions from *B. bifurcata* dichloromethane extract, and to investigate the relationships between TPC and the different antioxidant activity assays. The first two main components explained 49.5% and 34.1% of the total variance in the dataset, respectively (Figure 1). The horizontal axis expresses an opposition between DPPH (left) and TPC (right), verified in the first principal component (PC1) analysis. Moreover, TPC presented a negative correlation with DPPH, but showed a positive correlation with FRAP and ORAC (Figure 1). DPPH radical scavenging activity is expressed by IC_50_, and it is possible to verify that fractions presenting high phenolic content also exhibit high DPPH radical scavenging activity (Group I). On the other hand, F2 and F4 fractions showed low levels of TPC, and exhibited low DPPH radical scavenging activity (Group II) (Figure 1). However, F5 and F7 fractions showed high FRAP and ORAC values when compared with F3 and F6 fractions (the second principal component (PC2) explained the variation between samples with regard to peroxyl radical scavenging activity (ORAC) and ferric reducing power (FRAP).

In fact, the existence of a correlation between total phenolic content and antioxidant capacity has been reported by several authors [21,22,23]. According to the results obtained, it is possible to verify this relation in the PCA, in which the fractions that presented high total content of polyphenols also presented a high capacity of radical DPPH reduction. These data suggest that the observed relationship can be mediated by molecules such as polyphenols, which are good antioxidants because of their redox properties, which allow them to act as reducing agents [22,24], and hydrogen (H^+^) donors, contributing to the production of less reactive radicals, and for the removal of metal ions [25,26,27]. This mechanism plays an important role in preventing oxidation processes associated with the development of different pathologies such as neurodegenerative diseases [19]. In addition, previous studies have already demonstrated that *B. bifurcata* had interesting antioxidant activities, corroborating the results obtained in this study [10,19,27,28]. Among the compounds present in brown seaweeds, the phlorotannins are strong candidates to mediate those activities, since they are included in the major group of phenolic compounds, are abundant in brown algae, and are also reported for their enormous antioxidant potential. Li et al. [29] fractionated the brown algae *Sargassum fusiforme* with ethanol, ethyl acetate, and butanol for the extraction of phlorotannins, and found that the ethyl acetate fraction showed the highest phlorotannin content expressed in phloroglucinol equivalents (PGE) (884.8 mg PGE/g extract). From the ethyl acetate fraction, these authors isolated four phlorotannins, belonging to the classes of fuhalols, fucophlorethols, phlorethols, and eckols. Leyton et al. [28] studied the phlorotannin content of the brown alga *Macrocystis pyrifera*. They used different solvents, e.g., water, methanol, ethyl acetate, chloroform, and ethanol/water (70:30), the latter showing the highest total phlorotannin content (140 mg PGE/g extract). These studies are in agreement with the results obtained in our work, suggesting that the presence of phenolics, like polyphenols and phlorotannins, in *B. bifurcata* extracts and fractions, are responsible for their antioxidant properties. In recent years, antioxidant compounds, e.g., phenolics and other groups of compounds (diterpenes, polysaccharides, alkaloids) have shown promising therapeutic potential as agents that may delay the process associated with neuronal cell loss in neurodegenerative diseases such as PD [30,31,32,33].

### 2.2. Neuroprotective Effect of B. bifurcata on SH-SY5Y Cells Exposed to 6-OHDA

**Figure 2:**
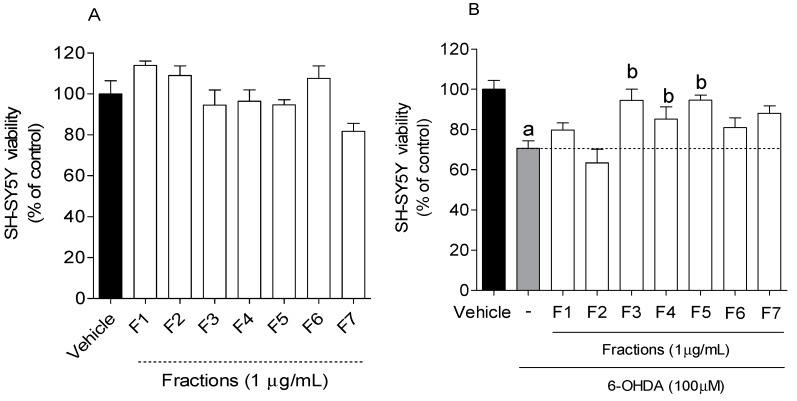
(**A**) Cytotoxicity of *B. bifurcata* fractions (1 µg/mL, 24 h) on SH-SY5Y cells; (**B**) Neuroprotective effects of *B. bifurcata* fractions (1 µg/mL) on SH-SY5Y cells during 24 h of incubation with 6-OHDA. The values in each column represent the mean ± standard error of the mean (SEM) of 3 or 4 independent experiments. Evaluation of statistical significance was performed using one-way ANOVA with Dunnett′s multiple comparison test (*p* value < 0.05). Symbols represent statistically significant differences when compared to: ^a^ vehicle. ^b^ to 6-OHDA.

The cytotoxic effects of *B. bifurcata* fractions on SH-SY5Y cells were evaluated. Fractions with no cytotoxicity were further tested for their neuroprotective potential on SH-SY5Y cells after exposure to the neurotoxin 6-OHDA. Cells were treated with *B. bifurcata* fractions (1 µg/mL) for 24 h, and cell viability was evaluated by the MTT method. The results are presented in Figure 2.

The exposition of SH-SY5Y cells to 6-OHDA (100 µM) led to a reduction of about 30% (70.61% ± 3.73% of viable cells) of cell viability after 24 h incubation when compared to vehicle (100 ± 4.43 of viable cells). However, when 6-OHDA was incubated with fractions (1 µg/mL), the F3, F4 and F5 fractions exhibited the ability to revert its neurotoxic effect on cell viability to about 20–25% (Figure 2). 

The decrease of neurotoxicity associated with 6-OHDA mediated by extracts and compounds has been reported by several scientific studies [2,9,34]. For instance, a previous study carried out by our group demonstrated the neuroprotective effect of several extracts of macroalgae in SH-SY5Y cells when exposed to 6-OHDA, inducing an increase of 15–35% of cell viability at a concentration of 1 mg/mL [9]. On the other hand, Ba et al. [35] found that co-treatment with schisandrin B, isolated from a plant, increased cell viability in 35% (40.05 μg/mL) in SH-SY5Y cells in the presence of 6-OHDA. Lou et al. [36] isolated naringenin from a fruit and found that co-treatment with this compound resulted in an increase in cell viability of 30% (21.78 μg/mL) in SH-SY5Y cells also treated with 6-OHDA. The neuroprotective effect demonstrated by these compounds was somewhat similar to that obtained with *B. bifurcata* fractions. However, the concentrations tested by the authors were higher (>1 μg/mL) when compared to the concentration used in this study. Therefore, the results obtained are quite promising, since these samples are crude fractions, which are still composed of a variety of molecules that, once isolated and purified, can have their in vitro neuroprotective effects potentiated.

### 2.3. Cellular Mechanisms Associated with the Neuroprotective Effect of B. bifurcata

**Figure 3:**
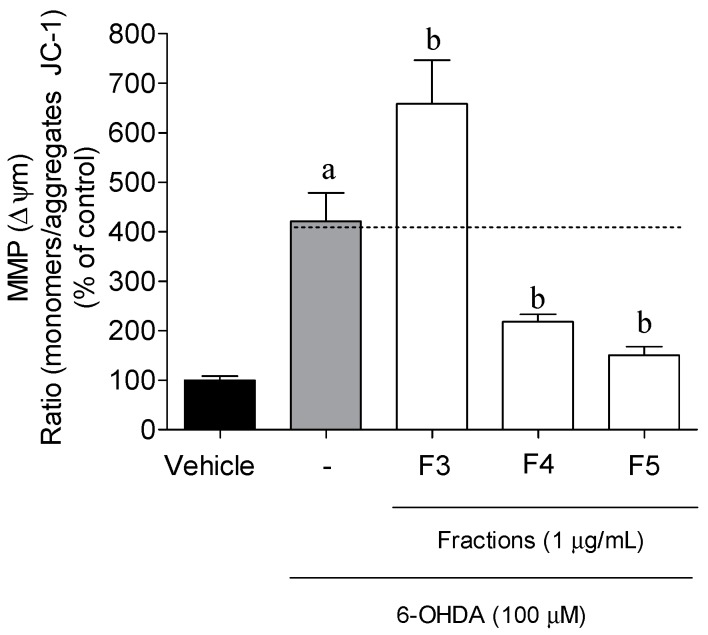
Mitochondrial membrane potential of SH-SY5Y cells after 6 h of incubation with 6-OHDA (100 µM) in the absence or presence of *B. bifurcata* fractions (1 µg/mL). Results were obtained by the ratio between the monomers/aggregates of JC-1. The values in each column represent the mean ± standard error of the mean (SEM) of 3 or 4 independent experiments. One-way ANOVA with Dunnett’s multiple comparison test was conducted to evaluate statistical significance (*p* value < 0.05). Symbols represent statistically significant differences when compared to ^a^ vehicle and ^b^ 6-OHDA.

**Figure 4:**
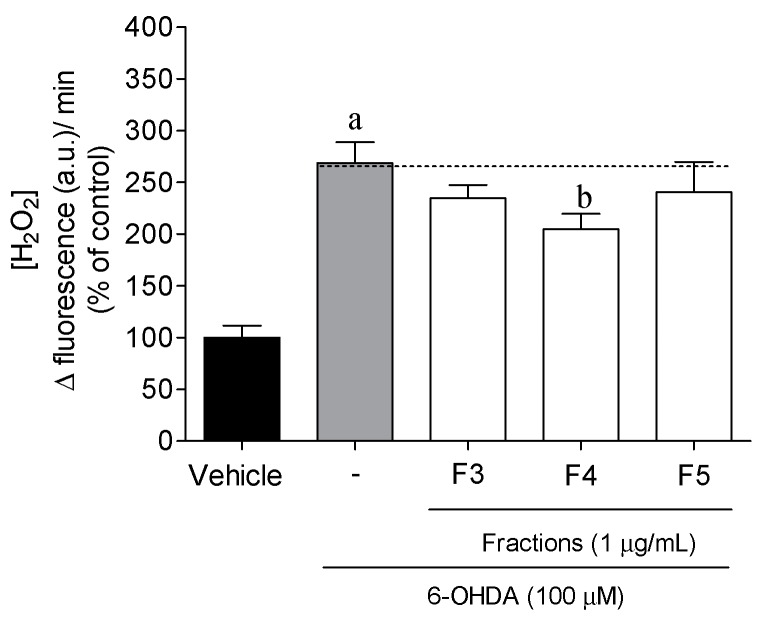
Levels of hydrogen peroxide (H_2_O_2_) produced by SH-SY5Y cells after 12 h of incubation with 6-OHDA (100 µM) in the presence or absence *Bifurcaria bifurcata* fractions (1 µg/mL). H_2_O_2_ was quantified fluorometrically using the Amplex Red Hydrogen Peroxide/Peroxidase Assay kit. The values in each column represent the mean ± standard error of the mean (SEM) of 3 or 4 independent experiments. One-way ANOVA with Dunnett′s multiple comparison test was conducted to evaluate statistical significance (*p* value < 0.05). Symbols represent statistically significant differences when compared to ^a^ vehicle and ^b^ 6-OHDA.

**Figure 5:**
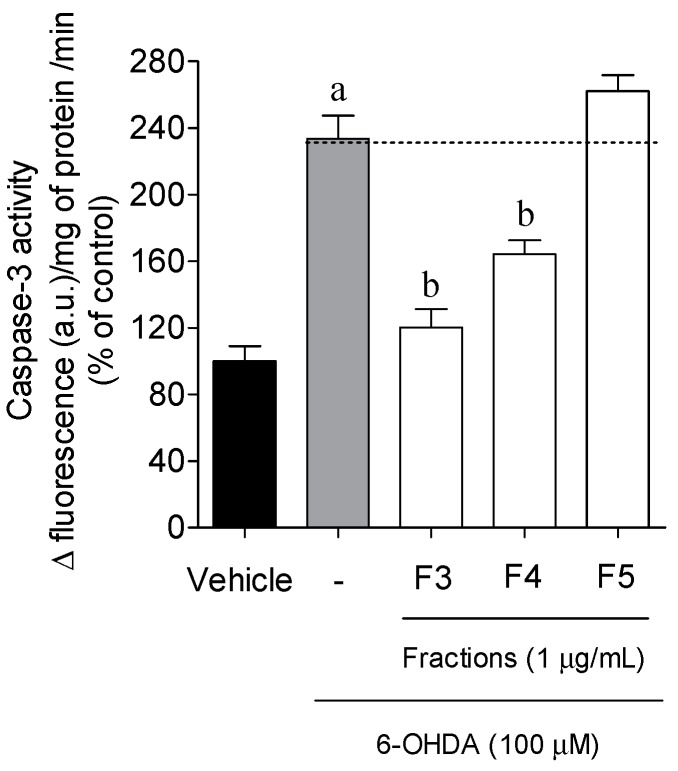
Effects of 6-OHDA (100 µM) in the absence or presence of *B. bifurcata* fractions (1 µg/mL) on Caspase-3 activity of SH-SY5Y cells after 6 h of treatment. The activity was quantified by the slope of the linear phase accumulation of rhodamine 110 (between 20 and 40 min). The results are presented in arbitrary units of fluorescence per mg protein per min. The values in each column represent the mean ± standard error of the mean (SEM) from 3 to 4 experiments. One-way ANOVA with Dunnett’s multiple comparison test was conducted to evaluate statistical significance (*p* value < 0.05). Symbols represent statistically significant differences when compared to ^a^ vehicle and ^b^ 6-OHDA.

**Figure 6:**
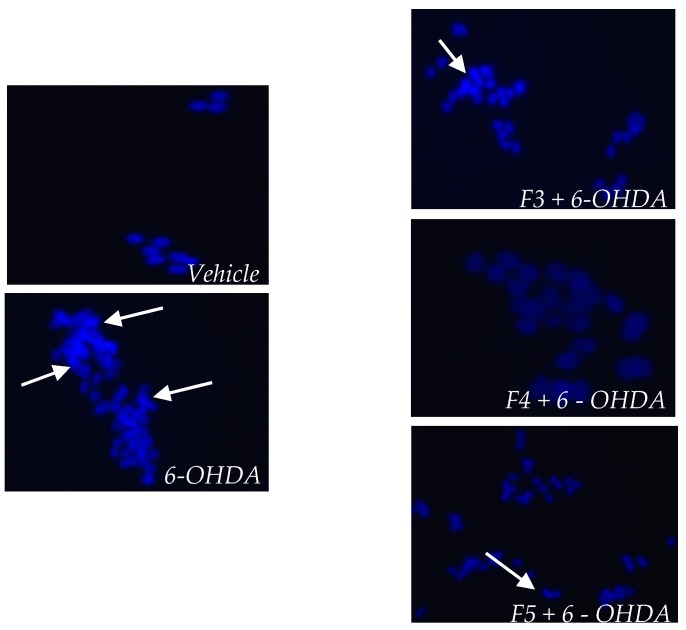
Nuclear morphology of SH-SY5Y cells stained with DAPI probe. SH-SY5Y cells stained with DAPI showing the anti-apoptotic effect of the fractions F3, F4 and F5 of *Bifurcaria bifurcata* (1 µg/mL) against neurotoxicity mediated by 6-OHDA (100 µM; 24 h). The fragmentation pattern is an indicator of apoptosis. * The images are representative of one experiment.

**Figure 7:**
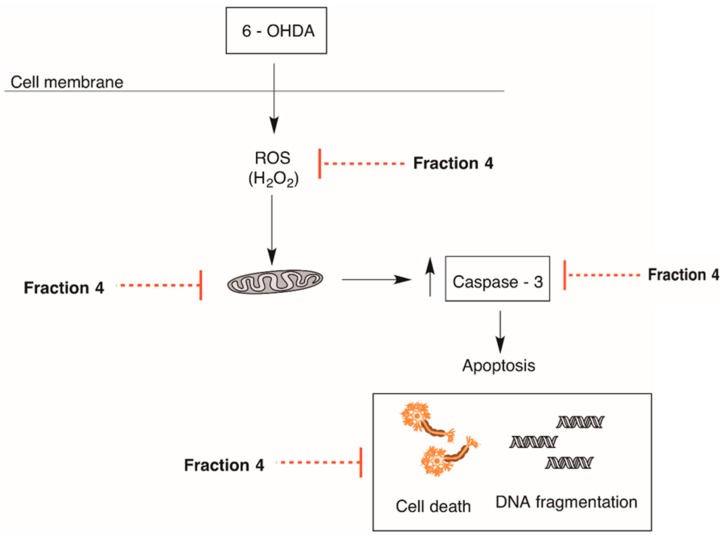
Schematic hypothesis of neuroprotective mechanism mediated by F4 fraction derived from *Bifurcaria bifurcata* on 6-OHDA-injured SH-SY5Y cells.

To investigate if the neuroprotective effect demonstrated by *B. bifurcata* on the viability of SH-SY5Y cells was associated with PD hallmarks, different in vitro assays were performed (mitochondrial membrane potential, production of H_2_O_2_, Caspase-3 activity, and DNA fragmentation) on cells treated with the neurotoxin 6-OHDA in the absence or presence of fractions from *B. bifurcata*. The incubation times were defined according to the highest effects promoted by 6-OHDA for each mechanism, verified in preliminary studies. The treatment with the F1–F7 fractions did not change the results of the mitochondrial potential, H_2_O_2_ production, and Caspase-3 activity when compared with the vehicle (cells without treatment), per se. However, for a clear understanding of the results, all the data were presented as percentage of control. The results are presented in Figure 3, Figure 4, Figure 5, Figure 6 and Figure 7.

The membrane mitochondrial potential (MMP) determination allowed us to measure the mitochondrial dysfunction and to understand if the neuro protective effect of *B. bifurcata* was mediated by biological events on mitochondria. The exposition of SH-SY5Y cells to 6-OHDA (100 µM, 6 h) induced a strong depolarization of the MMP when compared with the vehicle (*p* < 0.05) (Figure 3). During the treatment with seaweed fractions, it was possible to observe a noticeable preventive effect of F4 (218.10% ± 14.87% of vehicle) and F5 fractions (150.51% ± 16.98% of vehicle) in the depolarization induced by 6-OHDA (421.48% ± 57.05% of vehicle) exposition. On the contrary, F3 enhanced the mitochondrial dysfunction.

The levels of H_2_O_2_ production were measured to evaluate the ability of *B. bifurcata* fractions to prevent the condition of oxidative stress induced by the neurotoxin (Figure 4). SH-SY5Y cells, when exposed to 6-OHDA (100 μM, 12 h), experienced an increase of more than twice the H_2_O_2_ levels when compared with vehicle (*p* < 0.05). Moreover, when *B. bifurcata* fractions were administered at 1 µg/mL, only F4 showed a significant effect in the decrease of the levels of H_2_O_2_ (204.50% ± 15.12% of vehicle) as compared to 6-OHDA (268.73% ± 20.03% of the vehicle) treatment. 

Caspase-3 activity was measured to understand if the *B. bifurcata* fractions had capability to prevent the cell death mediated by apoptosis when exposed to the neurotoxin (Figure 5). The treatment of SH-SY5Y cells with 6-OHDA (100 µM, 6 h) (233.58% ± 13.78% of vehicle) has shown an increase of Caspase-3 activity compared with vehicle. Fraction F5 had an effect on this enzyme that was similar to 6-OHDA, while F3 (120.11% ± 11.00% of vehicle) and F4 (164.24% ± 8.31% of vehicle) fractions showed a decreased Caspase-3 activity when compared with 6-OHDA. 

To understand if *B. bifurcata* fractions had the ability to prevent the DNA fragmentation induced by 6-OHDA treatment, the integrity of SH-SY5Y DNA was evaluated through staining with DAPI probe. Cell death by apoptosis was also investigated through fluorescence microscopy after DAPI staining. Nuclear condensation and fragmentation, which are characteristic features of apoptosis, were found for neuroblastoma cells treated with 6-OHDA (100 µM, 24 h) (Figure 6). The obtained images are representative of one experiment. However, it was possible to verify that treatment with fractions of *B. bifurcata* can inhibit nuclear condensation and fragmentation, and the protective effect of F4 fraction against 6-OHDA-induced apoptosis was verified.

Overall, the data concerning the cellular mechanisms behind the neuroprotective activity of *B. bifurcata* suggest that the effect demonstrated by F4 fraction is mediated by a reduction of oxidative stress condition via H_2_O_2_ production pathway and an anti-apoptotic effect (mitochondrial protection, decrease of Ccaspase-3 activity, and decrease of DNA condensation) which has not been verified for F3 and F5 fractions. 

Mitochondria produce more than 90% of cellular energy through oxidative phosphorylation, which involves the Krebs cycle and the electron carrier chain. However, about 1% to 2% of the electrons are released through the complexes I and III of the electron transport chain, reacting with molecular oxygen and forming ROS sequentially as O_2_^−●^ and H_2_O_2_, which can be converted to water through the peroxisome. An increase of ROS levels in mitochondria may activate apoptotic pathways, leading to cell death. Thus, a blockade of the complex I can result in mitochondrial dysfunction culminating in several consequences, namely, the increase of ROS (H_2_O_2_) and a decrease of ATP production and cell damage [37,38]. Based on these facts, the decrease in the neurotoxic effect of 6-OHDA, evidenced by the F4 fraction, clearly appears to be associated with different mechanisms that are intercorrelated, in this case, to a decrease in H_2_O_2_ production and mitochondrial depolarization. These results are in agreement with those obtained by Cui et al. [39], which demonstrated that SH-SY5Y cells submitted to neurotoxic effects induced by 6-OHDA, and that treatment with a sulfated polysaccharide isolated from the sea cucumber *Stichopus japonicus* prevented changes in mitochondrial membrane potential, induced a reduction of ROS level, and increased cell viability. Kich et al. [40] also demonstrated that in SH-SY5Y cells treated with 6-OHDA, the presence of *Calyptranthes grandifolia* extracts prevented alterations in mitochondrial potential and reduced the levels of H_2_O_2_ production. Several studies have shown that neuronal death in PD is associated with the release of cytochrome C and, consequently, activation of the caspase cascade, namely caspase-3, which plays an important role in the process of cell death by apoptosis characterized by cellular shrinkage and nuclear condensation [41,42]. According to our results, it was possible to verify that the F4 fraction caused an inhibition of Caspase-3 and reversed the morphological damage induced by 6-OHDA. These results suggest that the increasing of cell viability may be mediated by an effect on the apoptosis process, which is in accordance with several studies in which the use of different molecules exhibited anti-apoptotic effects, namely by decreasing caspase and reversing the morphological damage caused by 6-OHDA [39,43,44]. In this study, the F4 fraction demonstrated the highest neuroprotective activity, which seems to be related with a decrease of oxidative stress condition via H_2_O_2_ production, and an anti-apoptotic effect as described before. These results led us to hypothesize the mechanism of action represented in Figure 7.

### 2.4. Isolation and Antioxidant Activity of B. bifurcata Compounds

**Figure 8:**
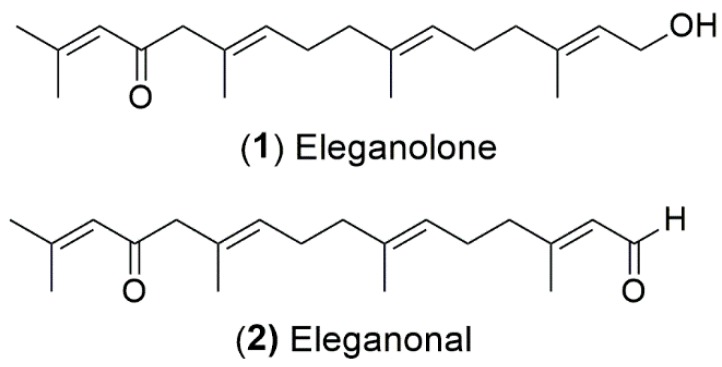
Structures of the compounds **1** and **2**.

Since the F4 fraction from *B. bifurcata* revealed the best neuroprotective activity, it was subjected to more refined fractionation by semi-preparative HPLC, aiming to isolate the potential bioactive molecules. Two major compounds, eleganolone (**1**) and eleganonal (**2**), were obtained (Figure 8), whereby the latter was obtained after further purification by preparative TLC.

The structures of both metabolites were established by nuclear magnetic resonance (NMR), and by comparison of their spectral data with those described in the literature [18,45]. 

Eleganolone was described by Ghothel et al. [18] and eleganonal was reported for the first time by Culioli et al. [46]. These two compounds belong to the family of diterpenes and are usually found in this species [17,18,45,46,47,48]. 

Although both compounds have already been described a few years ago, much remains to be explored regarding their biological potential. Only Gallé et al. [17] evaluated the anti-protozoal activity of eleganolone (**1**) against *Plasmodium falciparum* and *Leishmania donovani*, as well as its cytotoxic activity on mammalian L6 cell line, concluding that eleganolone could be the active compound against *P. falciparum*.

The antioxidant activity of eleganolone (**1**) and eleganonal (**2**) isolated from *B. bifurcata* was evaluated by the DPPH, ORAC, and FRAP methods to verify if both compounds contributed, or not, to the antioxidant activity presented by F4 fraction. The results are presented in Table 2. 

As can be seen in Table 2, both compounds did not demonstrate a significant ability to reduce the DPPH radical, when compared to the fraction from which they were isolated. However, the compounds showed a high potential in reducing peroxyl radicals and have a strong iron reduction capacity, when compared to the synthetic antioxidant BHT.

An increase in iron and other metal ions may be associated with neurodegenerative diseases, namely PD, in the *substantia nigra* (SNpar) region. During aging, the concentration of iron can change in the brain, leading to a metabolic stress condition [49,50]. Since SNpar has a high metabolic rate of dopamine, neuromelanin, iron, and low antioxidant content, such as reduced glutathione, lead to an increase of oxidative stress. The production of ROS thus results in mitochondrial alterations that will contribute to the disappearance of dopaminergic neurons [51]. In this way, the potential of both compounds, in particular eleganolone (**1**), in reducing iron may be interesting for future exploitation aiming at the prevention and/or treatment of PD. Currently, in vitro studies regarding the evaluation of the neuroprotective effects of both compounds are under development.

In conclusion, fraction F5 presented the best antioxidant potential in all the assays here described. However, fraction F4 revealed to have the highest neuroprotective effects which could be associated with the reduction of H_2_O_2_ production, protection of mitochondrial membrane potential, and inhibition of Caspase-3 activity. The compounds eleganolone (**1**) and eleganonal (**2**), obtained from this fraction, demonstrated antioxidant potential by FRAP and ORAC assays, revealing themselves to be excellent candidates for further neuroprotection assays aiming the prevention and/or treatment of PD. Currently, more studies on both compounds are being developed.

## 3. Materials and Methods

### 3.1. Chemicals and Reagents

Solvents for chromatographic techniques were HPLC quality grade (VWR, Fontenay-sous-Bois, France). VLC was performed on Kieselgel 60–200 µm (VWR, Ref. 84893.290, Leuven, Belgium), TLC on Kieselgel 60 F_254_ aluminium sheets (Merck, Ref. 5554, Darmstadt, Germany), were used for TLC while PTLC was performed on Kieselgel 60 F_254_ glass plates (Merck, Ref. 5744, Darmstadt, Germany).

For TPC determination, Folin reagent (Scharlau, Barcelona, Spain) and sodium carbonate (Panreac, Barcelona, Spain) were used. Antioxidant activity determination was accomplished by using the following reagents: TPTZ, FeCl_3_, and DPPH (Sigma, Steinheim, Germany), FeSO_4_ (Sigma, Karnataka, India), fluorescein, APPH, and Trolox (Sigma, St. Louis, MO, USA). 

Concerning the determination of neuroprotective activities, the following reagents were used: DMEM/F12, trypsin 1×, and FBS (Biowest, Riverside, MO, USA), antimicotic (Sigma, Rehovot, Israel), and MTT (VWR, Fontenay-sous-Bois, France). The hallmarks associated with oxidative stress and apoptosis were evaluated by using JC-1, Amplex Red Hydrogen Peroxide/Peroxidase Assay kit and DAPI (Molecular probes, Loughborough, UK), and Caspase-3 Activity Fluorimetric kit (Sigma, Rehovot, Israel).

### 3.2. Seaweed Collection 

Freshly samples of *B. bifurcata* were collected between May and June 2015, at Baleal Beach (39°37′68.32″ N 9°34′01.12″ W), Peniche (Portugal), and immediately transported to MARE-IPLeiria facilities. The samples were cleaned and washed with seawater to remove epiphytes, detritus, and encrusting material. After that, the clean material was freeze-dried (Scanvac Cool Safe, LaboGene, Lynge, Denmark), ground, and stored at −80 °C until further use.

### 3.3. Extraction and Fractionation of B. bifurcata

Freeze-dried samples of *B. bifurcata* (2.2 kg, 15% of wet seaweed), were sequentially extracted with MeOH and CH_2_Cl_2_ (in a biomass/solvent ratio of 1:40) at constant stirring for 12 h. Each resulting solution was filtrated, and the corresponding organic phase was evaporated under reduced pressure on a rotary evaporator (IKA HB10), at 40 °C. The CH_2_Cl_2_ extract (22 g, 0.95% of freeze-dried seaweed) was further concentrated and subjected to normal phase VLC on silica gel 60 (0.06–0.2 mm), with mixtures of cyclohexane/ethyl acetate (*v*/*v*) of increasing polarity, 1:0 (F1, 856 mg, 3.94%), 2:1 (F2, 5261 mg, 23.91%), 1:1 (F3, 3476 mg,15.80%), 1:2 (F4, 1550 mg, 6.84%), 0:1 (F5, 881 mg, 4.00%), MeOH (F6, 3942 mg, 17.92%) and acetone (F7, 887 mg, 4.03%) (500 mL of each eluent). The dichloromethane extract and the correspondent fractions were subjected to a series of in vitro studies, in order to evaluate their antioxidant and neuroprotective potential.

### 3.4. Isolation and Characterization of Bioactive Compounds 

Compounds from the most bioactive fraction (F4, 1550 mg) were isolated by semi-preparative HPLC (Jasco, LC—4000 series, Easton, EUA) by using a reverse-phase column (Synergi Fusion-RP 80 Åª, Phenomenex, 250 mm × 10 mm, 4 µm semi-preparative column). Compounds were eluted with a gradient of H_2_O/CH_3_CN: isocratic step from 0 to 5 min (50:50), then, a first gradient step from 5 to 15 min (50:50 to 100) (flow rate 2.0 mL·min^−1^, injection volume 200 µL). The first fractionation afforded 16 peaks (F4p1–F4p16), from which fraction F4p10, (t_R_ 27.5 min, 115.5 mg) was subjected to a more refined purification by PTLC over silica gel plates using *n*-hexane-ethyl acetate (7:3) as developing system, affording compounds **1** (38.8 mg; Rf = 0.44) and **2** (2.1 mg; Rf = 0.71).The structures of the two purified compounds were elucidated by nuclear magnetic resonance (^1^H, ^13^C, APT, COSY, HMBC and HSQC). NMR spectra were acquired on a Bruker Avance 400 spectrometer with a frequency of 400 MHz for ^1^H, and 100 MHz for ^13^C. Samples were dissolved in 500 µL of CDCl_3_ (Sigma-Aldrich, St. Louis, MO, USA). Chemical shifts were expressed in ppm and reported to the residual solvent signals. Coupling constants (*J*) are expressed in Hertz (Hz). 

**Eleganolone** (**1**): ^1^H NMR (400 MHz, CDCl_3_) δ 6.06 (1H, *s*, H-14), 5.35 (1H, *t*, *J* = 6.8 Hz, H-2), 5.19 (1H, *t*, *J* = 6.8 Hz, H-10), 5.06 (1H, *t*, *J* = 6.7 Hz, H-6), 4.09 (2H, *d*, *J* = 6.8 Hz, H_2_-1), 2.97 (2H, *s*, H_2_-12), 2.11 (3H, *s*, H_3_-17), 2.08 (2H, *m*, H_2_-9), 2.06 (2H, *m*, H_2_-5), 1.99 (2H, *m*, H_2_-4), 1.98 (2H, *m*, H_2_-8), 1.82 (3H, *s*, H_3_-16), 1.61 (3H, *s*, H_3_-20), 1.56 (3H, *s*, H_3_-18), 1.55 (3H, *s*, H_3_-19); ^13^C NMR (100 MHz, CDCl_3_) δ 199.55 (C-13), 155.68 (C-15), 139.58 (C-3),135.02 (C-7), 129.52 (C-11),129.10 (C-10), 123.92 (C-6), 123.34 (C-2), 122.80 (C-14), 59.33 (C-1), 55.35 (C-12), 39.46 (C-4), 39.29 (C-8), 27.71 (C-16), 26.70 (C-9), 26.21 (C-5), 20.66 (C-17), 16.36 (C-18), 16.24 (C-20),15.92 (C-19).

**Eleganonal** (**2**): ^1^H NMR (400 MHz, CDCl_3_) δ 9.92 (1H, *d*, *J* = 8.0 Hz, H-1), 6.10 (1H, *s*, H-14), 5.87 (1H, *d*, *J*= 8.0 Hz, H-2), 5.22 (1H, *t*, *J*= 6.2 Hz, H-10), 5.09 (1H, *t*, *J* = 6.2 Hz, H-6), 3.03 (2H, *s*, H_2_-12), 2.22 (2H, *m*, H_2_-4), 2.21 (2H, *m*, H_2_-5), 2.17 (3H, *s*, H_3_-20, 2.14 (3H, *s*, H_3_-17), 2.12 (2H, *m*, H_2_-9), 2.02 (2H, *m*, H_2_-8), 1.88 (3H, *s*, H_3_-16), and), 1.61 (6H, *s*, H_3_-18 and H_3_-19); ^13^C NMR (100 MHz, CDCl_3_) δ 199.49 (C-13), 191.32 (C-1), 163.87 (C-3),155.66 (C-15), 136.30 (C-7), 129.75 (C-11),128.90 (C-10), 127.40 (C-2), 122.86 (C-14), 122.64 (C-6), 55.34 (C-12), 40.56 (C-4), 39.28 (C-8), 27.73 (C-16), 26.68 (C-9), 25.62 (C-5), 20.68 (C-17), 17.60 (C-20),16.40 (C-18), 16.05 (C-19).

### 3.5. Antioxidant Activity 

Total Phenolic Content (TPC): the quantification of TPC on *B. bifurcata* samples was achieved using the Folin–Ciocalteu method adapted to a microscale [52], with minor modifications, and as described by Pinteus et al. [10]. Briefly, 2 µL of samples were added to 158 µL of distilled water in a 96-well microplate, followed by the addition of 10 µL of Folin–Ciocalteu reagent. The reaction mixture was pre-incubated for 2 min at room temperature, and then 30 µL of 20% Na_2_CO_3_ (w/v) was added and mixed. After 1 h of reaction in the dark, the absorbance was measured at 755 nm (Synergy H1 Multi-Mode Microplate Reader, BioTek^®^ Instruments, Winooski, VT, USA). Gallic acid was used as standard. TPC was expressed in milligrams of gallic acid equivalents per gram of dry extract (mg GAE/g of extract). 

DPPH Radical Scavenging Activity: this assay was performed according to Pinteus et al. [10]. An aliquot of 2 µL of *B. bifurcata* samples of different concentrations was added to 198 µL of DPPH ethanol solution (0.1 mM) to obtain final concentrations of 10, 30, 100, 300 and 1000 μg/mL and the mixtures were then incubated in the dark for 30 min at room temperature. Results are expressed as mean values ± SEM (standard error of the mean). IC_50_ values (μg/mL) were also determined for the extracts with highest activity (DPPH reduction >50%).

Oxygen Radical Absorbance Capacity (ORAC-fluorescein): this assay followed the methodology described by Dávalos et al. [53]. The reaction was carried out in 75 mM phosphate buffer (pH 7.4), and the final reaction mixture was 200 µL. *B. bifurcata* samples (20 µL), and fluorescein (120 µL; 70 nM, final concentration) were placed in the wells of the 96-well microplate. The mixture was pre-incubated for 15 min at 37 °C. Then, AAPH solution (60 µL; 12 mM, final concentration) was added rapidly, using a multichannel micropipette. The microplate was immediately placed in the reader and the fluorescence (λ_excitation_: 458 nm; λ_emission_: 520 nm), recorded every minute for 240 min, and automatically shaken prior to each reading. A blank using phosphate buffer instead of the fluorescein and eight calibration solutions, using Trolox (0–80 µM) as antioxidant standard, were also carried out in each assay. ORAC values were expressed as Trolox equivalents by using the standard curve calculated for each assay. Final results were expressed in µmol of Trolox equivalents/g of dry extract (or compound) (µmol TE/g of extract (or compound)).

Ferric Reducing Antioxidant Power (FRAP): this method was performed according to Benzie and Strain [54] and Li et al. [29], adapted to a microscale with minor modifications. FeSO_4_ was used as standard. FRAP reagent was prepared with 0.3 M acetate buffer (pH = 3.6), 10 mM of TPTZ in 40 mM HCl and 20 mM ferric solution using FeCl_3_ in a 96-well microplate. By freshly mixing acetate buffer, TPTZ, and ferric solutions at a ratio of 10:1:1, the final working FRAP reagent was incubated at 37 °C. Briefly, 2 µL of *B. bifurcata* samples were added to 198 µL of FRAP reagent and allowed to stand at 37 °C in the dark by 30 min, at which time the increase in absorbance at 593 nm was measured in the microplate reader. The difference between the absorbance of test compounds and the blank reading was calculated and expressed as µM of FeSO_4_/g of extract (or compound).

### 3.6. Mammalian Cell Strain and Culture Method

The experiments were performed in an in vitro model of human neuroblastoma SH-SY5Y cells, acquired in the DSMZ (Deutsche Sammlung von Mikroorganismen und Zellkulturen GmbH, strain number ACC 209). Culture conditions were performed according to DSMZ Bank. Briefly, SH-SY5Y cells were cultured in Dulbecco’s Modified Eagle’s Medium (DMEM) (Sigma-Aldrich, Munich, Germany) supplemented with 10% (*v*/*v*) of fetal bovine serum (FBS) (Biowest, Riverside, MO, USA) and 1% of antibiotic/antimycotic commercial solution (Sigma, Rehovot, Israel). For subculture, SH-SY5Y cells were dissociated with trypsin-EDTA (Biowest, Riverside, MO, USA), split into a 1:3 ratio, and subcultured into Petri dishes with 25 cm^2^ growth area. Culture medium was replaced every 2 days until the cells reached confluence 4–5 days after the initial seeding. Cells were maintained under controlled conditions in a 95% humidified atmosphere, 5% CO_2_, and constant temperature of 37 °C.

### 3.7. Neuroprotective Activity 

The neuroprotective effects of *B. bifurcata* fractions on SH-SY5Y cells treated with 6-OHDA was evaluated as previously described by Silva et al. [9]. Cells were incubated with different fractions of *B. bifurcata* [F1–F7] in the presence of 6-OHDA at a concentration of 1 µg/mL for 24 h. The solutions were previously prepared in culture medium without FBS and sterile-filtered (0.2 µm, Whatman, Maidstone, UK). The effects were assessed by a colorimetric assay based on the conversion of tetrazolium salts (MTT) to blue formazan products by active mitochondria [55]. 

### 3.8. Mechanisms Associated with Neuroprotective Potential 

Quantification of Hydrogen Peroxide (H_2_O_2_): This assay was performed using the Amplex Red Hydrogen Peroxide/Peroxidase Assay kit (Life Technologies, Camarillo, CA, USA), as previously described by Silva et al. [9]. The H_2_O_2_ production was quantified in SH-SY5Y cells after 12 h of treatment with 6-OHDA (100 µM) in the absence or presence of fractions (1 µg/mL) from *B. bifurcata*. The variation of H_2_O_2_ production was accompanied in real-time for 60 min at room temperature. Results were expressed in percentage of control. 

Mitochondrial Membrane Potential (MMP): MMP was determined using the fluorescent probe, JC-1 (Molecular Probes, Eugene, OR, USA), according to the method described by Silva et al. [9]. The SH-SY5Y cells were treated with 6-OHDA (100 µM) for 6 h, in the absence or presence of fractions (1 µg/mL) from *B. bifurcata*. Results were expressed as the ratio of the monomers/aggregates of JC-1 in percentage of control.

Caspase-3 Activity: This activity was assessed using the Caspase-3 Activity Fluorimetric kit (Sigma, Saint Louis, MO, USA), as described previously by Silva et al. [9]. Cells (SH-SY5Y) were cultured in 6-well plates and treated with 6-OHDA (100 µM) for 6 h in the presence or absence of fractions from *B. bifurcata* (1 µg/mL). Caspase-3 activity was calculated by the slope of the linear phase of the fluorescence resulting from the rhodamine 110 accumulation and expressed in arbitrary fluorescence units per mg protein per minute (Δ fluorescence (a.u.)/mg protein/min).

DAPI Staining: The nucleic condensation was determined by 4,6-diamidino-2-phenylindole (DAPI) staining (Applichem, Darmstadt, Germany). SH-SY5Y cells were cultured in 6-well plates and treated with 6-OHDA (100 µM) for 24 h in the presence or absence of *B. bifurcata* fractions (1 µg/mL). The cells were fixed in paraformaldehyde (4%) for 30 min. After this time, it was removed, and cells incubated in Triton X-100 (0.1%) for 30 min. Then, Triton X-100 was removed, followed by the addition of DAPI (1 µg/mL) solution. After 30 min reaction, DAPI was removed, and 1 mL PBS (pH 7.4) was added to each well. Then, the cells were observed through a fluorescence microscope (Zeiss, Axio Vert. A1, Oberkochen, Germany).

### 3.9. Statistical Analysis

One-way analysis of variance (ANOVA) with Dunnett′s multiple comparison of group means were employed to determine significant differences relatively to the control treatment. All other post hoc analyses were conducted using Tukey′s test. All data were checked for normality and homoscedasticity. Comparisons concerning variables, which did not meet variance or distributional assumptions, were carried out with Kruskal–Wallis non-parametric tests. When applicable, results are presented as mean ± standard error of the mean (SEM). Differences were considered statistically significant at a level of 0.05 (*p* < 0.05). All calculations were performed using IBM SPSS Statistics 24 (IBM Corporation, Armonk, NY, USA) and GraphPad v5.1 (GraphPad Software, Inc. La Jolla, CA, USA). The determination of IC_50_ was calculated by the analysis of non-linear regression using GraphPad Prism software with the equation Y = 100/(1 + 10 ^(X − LogIC_50_^^)^). PCA allowed for the detection of similarities and differences between the different samples, as well as the identification of the main associations between variables that are responsible for the total variability of the data studied. All calculations and graphs produced for the PCA study were performed using the software CANOCO for Windows 4.5 [56].

## Figures and Tables

**Table 1:** Antioxidant activity of dichloromethane extract and fractions from *B. bifurcata* collected in Peniche coast, Portugal.

Sample	TPC ^a^	DPPH ^b,^*	ORAC ^c^	FRAP ^d^
Crude Extract	10.63 ± 1.40	43.34 (36.90–50.81)	1886.55 ± 60.57	95.83 ± 4.48
F1	4.4 ± 0.60	111.9 (80.40–155.70)	353.23 ± 9.89	105.02 ± 1.88
F2	5.3 ± 0.60	>1000	768.01 ± 44.64	221.37 ± 11.77
F3	3.08 ± 0.30	>1000	412.55 ± 14.44	532.32 ± 3.00
F4	2.06 ± 0.41	>1000	1407.01 ± 44.56	548.19 ± 1.44
F5	44.14 ± 1.30	49.73 (31.56–78.56)	4469.14 ± 147.07	1128.20 ± 20.25
F6	7.26 ± 0.50	64.28 (40.59–101.80)	334.15 ± 49.65	7.64 ± 1.63
F7	9.82 ± 0.80	114.60 (40.24–118.40)	2861.25 ± 38.92	573.44± 33.79
BHT	-	205.00 (166.00–253.30)	143.70 ± 23.36	2821.50 ± 63.03

^a^ Gallic acid equivalents/extract (mg GAE/g); ^b^ radical scavenging activity (IC_50_ µg/mL); ^c^ Trolox equivalents/extract (µmol TE/g); ^d^ µM FeSO_4_/g extract. * IC_50_ values were determined for a 95% confidence interval.

**Table 2:** Antioxidant activity of compounds eleganolone (**1**) and eleganonal (**2**) isolated from *B. bifurcata* F4 fraction.

Compounds	DPPH ^a^	ORAC ^b^	FRAP ^c^
Eleganolone (**1**)	>100	1663.83 ± 25.35	8341.18 ± 177.72
Eleganonal (**2**)	>100	667.48 ± 10.96	8635.37 ± 389.54
BHT	>100	143.70 ± 23.36	2821.50 ± 63.03

^a^ Radical scavenging activity (IC_50_ µM); ^b^ µmol TE/g of compound; ^c^ µM FeSO_4_/g compound.
